# Social health, activity behaviors, and quality of life among young adult cancer survivors: Protocol for a prospective observational study

**DOI:** 10.1371/journal.pone.0309924

**Published:** 2024-11-08

**Authors:** Kimberly A. Miller, Jonathan N. Kaslander, Julia Stal, Britni R. Belcher, Junhan Cho, David R. Freyer, Kayla de la Haye, Joel E. Milam, Sarah E. Piombo, Maureen Cairns, Micaela Hewus, Priscilla Marin, Gino K. In

**Affiliations:** 1 Department of Population and Public Health Sciences, Keck School of Medicine of USC, Los Angeles, CA, United States of America; 2 Department of Pediatrics, Keck School of Medicine of USC, Los Angeles, CA, United States of America; 3 Cancer and Blood Disease Institute, Children’s Hospital Los Angeles, Los Angeles, CA, United States of America; 4 Department of Medicine, Keck School of Medicine of USC, Los Angeles, CA, United States of America; 5 Center for Economic and Social Research, Dornsife College of Letters Arts and Sciences, University of Southern California, Los Angeles, CA, United States of America; 6 Department of Epidemiology and Biostatistics, University of California, Irvine, Irvine, CA, United States of America; PLOS: Public Library of Science, UNITED KINGDOM OF GREAT BRITAIN AND NORTHERN IRELAND

## Abstract

Approximately 85,000 adolescent and young adults (AYAs; age 15–39) are diagnosed with cancer in the United States annually. Experiencing a cancer diagnosis as an AYA can substantially impact social connections and social health. This paper describes the design and protocol of an observational study to prospectively assess social health and its association with physical activity and quality of life among AYAs after a cancer diagnosis. The study uses a longitudinal observational design to prospectively explore the relationships between social health and activity behaviors (physical activity and sedentary time) at four clinically significant timepoints over the course of 12 months among AYAs newly diagnosed with cancer. Patients are recruited at three hospitals and surveyed at each time period. Multiple dimensions of social health are assessed including social support, social roles, loneliness, social anxiety, and social networks. A wrist accelerometer is worn for one week at each assessment period. Change in social network structures will be analyzed using egocentric social network analysis. Structural equation models will be fitted to analyze the relationship between social constructs and physical activity. Findings from this study will address gaps in our understanding of the impact of a cancer diagnosis on multiple dimensions of social health for AYAs and the potential role social factors play in physical activity and quality of life. Understanding these processes will inform age-tailored interventions to improve health and quality of life outcomes for this at-risk population.

## Background

Approximately 85,000 adolescent and young adults (AYAs; diagnosed age 15–39) are diagnosed with cancer in the United States annually [1]. Experiencing a cancer diagnosis as an AYA can lead to substantial emotional and physiological challenges due to age and life stage. AYA cancer survivors experience delays in diagnosis, limited access to cancer treatment, increased treatment-related toxicities, chronic medical conditions, high rates of depression and anxiety, fatigue, pain, impaired sleep and cognition, financial stress, among other physical and psychological challenges [2–9]. Such cancer-related impacts experienced at any point during the cancer trajectory can profoundly affect quality of life for this population.

Social challenges are frequently reported among AYAs as they face a life-threatening disease at a critical time in their development. AYAs experience difficulties forming new social relationships and maintaining existing ones following diagnosis, and report fewer than half as many social activities as their peers without a cancer history post-therapy [2, 10–15]. This lack of social connection and increased social stress following a cancer diagnosis often leads to adverse emotional experiences (i.e., loneliness) and can limit access to social support, a resource intimately connected to health [4, 14].

Social isolation and limited social support can compromise health through physiological and behavioral mechanisms including reduced engagement in healthy behaviors such as physical activity [16, 17]. Physical activity during treatment and in post-treatment survivorship is related to better health outcomes for cancer patients, including improved overall survival, reduced risk of secondary cancers, reduced risk of cancer-related disability and comorbid conditions, and better quality of life [4, 18–30]. Social factors may influence physical activity through the provision (or lack) of social support, such as information, encouragement, opportunities, or other resources that help individuals to be active [31]. Social factors may also indirectly influence health behaviors like physical activity by buffering the negative impacts of life stressors and reducing risk for negative outcomes like depression or substance use, which in turn can be barriers to healthy behaviors like physical activity [32]. Both social health and physical activity have been found to positively impact quality of life among cancer survivors [33]. Despite evidence that social health is central to AYA health outcomes, the complex changes in the social profiles of AYAs following a cancer diagnosis and their impact on physical activity and quality of life are not well understood.

Social health encompasses the diverse social phenomena that impact health and health behaviors including *social connections*, *social support*, and *social network structures*. Social connection and social support can be measured by an individual’s experiences of these phenomena. A social network lens yields an understanding of how social functions play out in the broader social structures that AYAs are embedded within. Both physical activity and inactivity have been associated with social network processes and features including *homophily*, or connections with similar others, who provide exposure to social norms and influence; *density*, or the number of social connections within a person’s social network; and *centrality*, whether an individual in the network occupies a highly connected and influential structural position [34–37].

The purpose of this manuscript is to describe the design and protocol of an observational study to prospectively assess the construct of social health and its influence on physical activity, sedentary time, and quality of life for AYA cancer patients. In this study, social health among AYAs will be assessed both from an *individual perspective*, reflecting AYA’s personal experience of their social context (e.g., perceived social belonging, connection, and support) and a *social-structuralist perspective* (features of the structure, composition, and function of their social networks. These social phenomena will be assessed at clinically significant timepoints (at baseline near to diagnosis, mid-treatment, near end of treatment, and 12-month follow-up) throughout the cancer trajectory. Overall, this study aims to characterize trajectories of social health; to investigate the longitudinal and potential reciprocal associations between social health and activity behaviors; and to identify the sociodemographic and clinical moderators of these relationships in AYA cancer patients. Study hypotheses are: 1) while social health trajectories will be heterogeneous, there will be an *overall decline* in social health during the assessment period, and these declines in social health during the course of therapy will predict lower quality of life in survivorship; 2) higher levels of social health will be associated with increases in physical activity and decreases in sedentary time, and the reciprocal relationships between social health and activity behaviors will be associated with quality of life in survivorship; and 3) gender, race/ethnicity, socioeconomic status (SES), and health status (symptom burden) will moderate the relationship between social health, activity behaviors, and their association with quality of life outcomes.

## Materials and methods

### Study setting

The study takes place at three hospitals affiliated with the University of Southern California (USC) in Los Angeles (LA), California: USC Norris Comprehensive Cancer Center (National Cancer Institute (NCI) designated comprehensive cancer center), Keck Hospital of USC (private, not-for-profit academic medical center), and Los Angeles General Medical Center (large public safety net and teaching hospital owned by LA County and staffed by USC doctors). All serve LA County, which as of 2023 has a population of 10 million people and a majority-minority population (49.1% Hispanic, 25.3% non-Latino White, 15.6% Asian, 9% African American) [38].

### Overview of study design

**Fig 1:**
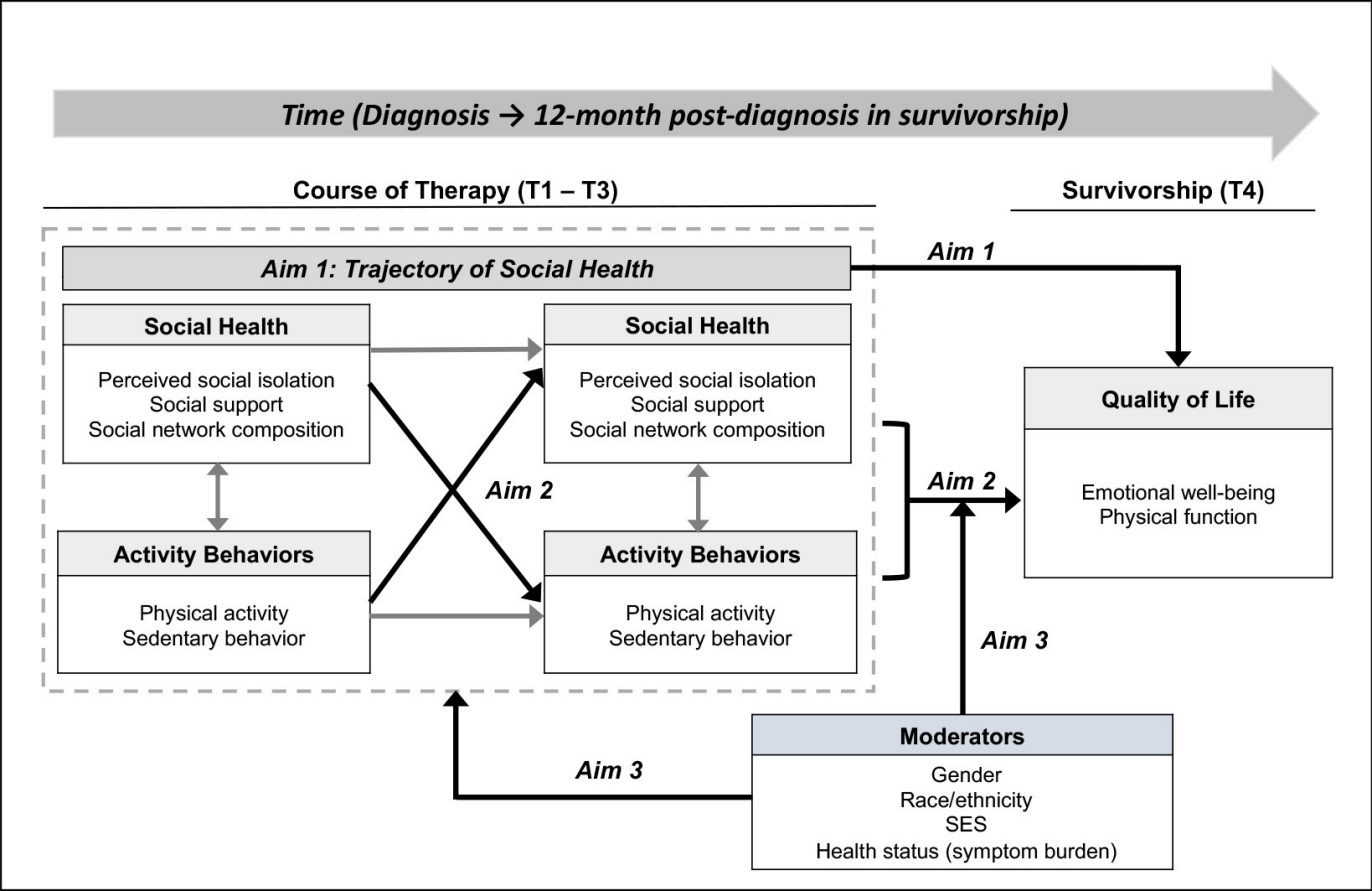
Conceptual model.

This study is a longitudinal observational design. A conceptual model guiding this study is shown in **Fig 1**. We will prospectively explore the relationships between social health and activity behaviors (physical activity and sedentary time) throughout cancer treatment and into post-treatment survivorship among ethnically and racially diverse AYAs with cancer. We will also explore potential moderators—sex, race/ethnicity, socioeconomic status (SES), and symptom burden—to identify specific sociodemographic characteristics that might put patients at higher risk for poorer social health.

Participants respond to self-administered electronic questionnaires about perceived social support and social connection, social networks, symptom burden, and quality of life over four timepoints: within three months of diagnosis, mid-treatment (3-month post-baseline), near end of treatment (6-month post-baseline), and 12-month follow-up (post-therapy survivorship for most patients). Patients’ activity behaviors will be assessed via a wrist-worn accelerometer worn for one week during each assessment period. The study was approved by the University of Southern California Institutional Review Board (IRB) before recruitment began. A partial Health Insurance Portability and Accountability Act (HIPAA) waiver was granted to identify patients in the electronic medical record (EMR) and the study was approved for written informed consent from participants for study participation as well as a signed HIPAA Authorization form for accessing medical records.

### Participants and recruitment methods

#### Study sample and stratified sampling plan

The study sample comprises patients with cancer types that are prototypical for AYAs and the most common within LA County, including the following cancer sites and International Classification of Diseases [ICD] codes: testis (C62), breast (C50), thyroid (C73), cervix (C53), colorectal (C18-20), brain/central nervous system (C71), non-Hodgkin’s lymphoma (C82-86), uterine (C54-55), melanoma (C43), bones and joints (C40-4), ovary (C56), Hodgkin’s lymphoma (C81), lung (C34) and gastric (C16).

Inclusion criteria consist of 1) aged 18–39 and diagnosed with the aforementioned cancer types within the past three months; 2) cancer stage I-IV for solid tumors as per American Joint Committee on Cancer (AJCC) 8^th^ edition TNM Classification of Malignant Tumors (TNM) staging, or I-IV for lymphomas as per Ann Arbor staging; 3) in care at one of the three USC hospitals at baseline; and 4) an expected survival of greater than one year at time of diagnosis (per clinician judgement). Leukemias are excluded as many of these are associated with longer duration of treatment. Exclusion criteria include having a primary language other than English or Spanish, or an inability to complete a survey or wear an accelerometer. Reasons for exclusion are identified when approaching participants, from the medical chart, and/or in consultation with clinicians.

Based on data from a pilot study led by the Principal Investigator among the target population in USC hospitals, we conservatively anticipate a minimum recruitment rate of 60% and an overall attrition rate of 15–20%. We will aim to recruit 200 participants over a three-year period with approximately 60% of Hispanic/Latino individuals as per the case mix of USC hospitals. To achieve a representative sample of the distribution of cases and to avoid oversampling more prevalent cancers, we will recruit the approximate percentage of cases of each cancer type that contributes to the overall eligible cases (e.g., thyroid accounts for approximately 14% of cases treated at USC hospitals; therefore, we will recruit 27 participants [i.e., 14% of 200]). **Table 1** shows the total number of cases seen annually at USC hospitals that are eligible for study screening and the stratified sampling scheme used.

**Table 1:** Eligible cases for recruitment at target hospitals 2016–2017 and stratified sampling scheme.

*Cancer type *	*Total patients*	*Patients selected for recruitment*	*Percent of cases to total eligible*
Testis	60	35	18%
Thyroid	45	27	14%
Breast	30	18	9%
Colorectal	28	18	9%
Brain/CNS	28	15	9%
Uterine	25	15	8%
Cervix	24	15	7%
HL	20	13	6%
Bones and joints	18	11	6%
Melanoma	17	10	5%
Gastric	11	7	3%
Ovary	7	6	2%
NHL	6	5	2%
Lung and bronchus	6	5	2%
Total	**325**	**200**	**100%**

### Case identification

Participants are identified within the past three months of a *de novo* cancer diagnosis through a data report from the EMR using International Classification of Diseases (ICD) codes to capture diagnoses new to the hospital. Each case contained in the reports is manually evaluated in the EMR based on eligibility criteria and checked to ensure that the participant does not have a previous diagnosis and has transferred their care from another medical setting or is a patient with recurrent disease. Participants are also identified through contacts with clinicians or at tumor boards for cancer types eligible for inclusion in this study. For all stage IV participants, study recruiters consult with the PI and the cancer care team before approaching the patient, including confirmation of life expectancy greater than 12 months. For participants who are newly diagnosed (e.g., within the first several days of their diagnosis), a 30 day waiting period is employed before contact to reduce burden.

### Participant tracking

A custom HIPAA-compliant Research Electronic Data Capture (REDCap) database housed at Southern California Clinical and Translational Science Institute (SC CTSI) is used to maintain all participant data including contact information, call logs, relevant dates and milestones, and incentive payments. All questionnaire responses and activity data are linked through this database and tracked via assigned ID numbers to preserve confidentiality. Study accrual totals, cancer types, demographic information and other relevant tracking information are aggregated to an online dashboard for the purposes of study oversight, recruitment targeting, and annual reporting.

### Recruitment methods

Eligible participants are approached by one of two recruitment specialists (one recruiter is bilingual and primarily covers Los Angeles General, which has a high proportion of Spanish-speaking patients). An initial introductory letter is sent to the patient describing the study aims and expected activities. Four days following this letter, recruiters attempt contact via phone, email, or text message. Three rounds of contact separated by seven days are attempted before considering the patient to be non-responsive. Calls and texts are made through Google Voice to allow for centralized collaborative communications, to maintain a call and text message record for each participant, and for a single phone number point of contact for participants. Contacts are stored in Google Voice as study identification numbers for confidentiality purposes. Contact logs are maintained in the REDCap tracking database which provides calculated dates for subsequent contacts and reports are used to track recruiters’ contact with participants.

Once a patient agrees to participate, the recruiter follows an established script to explain study procedures, activities, incentives, and the consent process. This occurs in English or Spanish, determined by the participant’s language of choice. Participants are then sent a link to the REDCap consent form and survey by email or text. A date for accelerometer wear is established and they are mailed the accelerometer and incentive payment card. Participants are compensated $50 for completing each assessment period plus a $50 bonus for finishing all four waves of assessment for a total of $250.

At each stage of the recruiting and study process, a status field in the database is updated. These statuses (i.e., active, completed, declined, withdrew, lost to follow up, and no response) allow the study team to maintain accurate accrual totals, understand the current state of the enrolled cohort, and track recruiting efforts. In addition, cancer type, gender, ethnicity, treating hospital, and other demographic and study status information (such as date of incentive payments and completion of HIPAA forms) are monitored in real time to ensure that the desired participant ratio and study milestones are achieved.

### Consent

Written informed consent for participation is done via REDCap forms online that are archived digitally and available for download by the participant. At the third wave of surveys (6 months), participants are informed about the study protocol to examine medical records and provided with a HIPAA consent form to allow access. Consenting to this component of the study is not required for participation.

### Medical chart abstraction

Chart abstraction will occur once a participant completes the study to extract and validate cancer treatment and symptoms in detail. Clinical factors drawn from participants’ medical charts include date of diagnosis, cancer site, stage at diagnosis, pertinent histology, treatment received (chemotherapy, immunotherapy, radiation, surgery, etc.), relapse/progression of disease, and vital status along with cause of death when applicable. Medical chart abstraction will be compared to self-report to ensure validity. If differences exist, clinical data from the EMR will be used in analyses. For participants who do not consent to HIPAA authorization, self-reported variables will be used. Consistent with the Principal Investigator’s prior pilot study, we anticipate an 80% response rate for HIPAA authorization.

### Accelerometer data and processing

At each assessment wave, patients are provided with a wrist-worn Actigraph GT3X-BT accelerometer and asked to wear it for one full week (Wednesday to Tuesday, activating at 6 am on the first day through 11 pm on the final day) to capture both weekend and weekday activity behaviors. The monitor is programmed to record at 80Hz. Participants are provided with instructions for proper positioning on their non-dominant wrist. The wrist strap is removable for situations such as medical procedures, but participants are asked to keep the accelerometers on at all times for seven days. At the end of the assessment period, participants return the monitor via mail. The accelerometer does not display any feedback to participants, and they are not informed of their activity levels during or after the assessment periods so as not to influence activity patterns.

The ActiLife software is used to download the data and to check for participant compliance or errors. Raw data files are converted from proprietary.agd format to.csv format and securely archived for later analysis. The raw data are converted into monitor-independent movement summary (MIMS) units, following the Belcher et al. protocol [39]. Total activity is represented by accumulated daily MIMS units, averaged across all days of wear.

### Survey measures

**Table 2** lists the name and source of the survey measures used. All questionnaire items are available in English and Spanish.

**Table 2:** Measures.

MEASURE	BASELINE	3 MONTHS	6 MONTHS	12 MONTHS
***Demographics***				
Self-report of current age, race/ethnicity, income level, educational level, marital status, and health insurance status	X			X
***Social Health***				
PROMIS Ability to Participate in Social Roles and Activities Short Form 4a [53]	X	X	X	X
PROMIS Emotional Support Short Form 4a [53]	X	X	X	X
PROMIS Informational Support Short Form 4a [53]	X	X	X	X
PROMIS Instrumental Support Short Form 4a [53]	X	X	X	X
Mini Social Phobia Inventory (MINI-Spin) [54]	X	X	X	X
UCLA Loneliness Scale short form [55]	X	X	X	X
Social Network survey (developed for this study using standard measures)	X	X	X	X
Cancer support group participation [56]	X	X	X	X
***Psychological measures***				
Eight-Item Center for Epidemiologic Studies Depression Scale (CES-D 8) [57]	X	X	X	X
Extraversion (Big Five Inventory) [58]	X			
Body Image Scale (adapted) [59]	X	X	X	X
***Quality of life***				
PROMIS Global Health-10 [60]	X			X
***COVID Impact***				
Pre- and post-pandemic physical activity and social health questions (adapted) [61]	X			
***Physical Activity (self-report)***				
International Physical Activity (IPA Questionnaire)	X	X	X	X
***Symptom Burden***				
MD Anderson Symptom Index (MDASI) [62]	X	X	X	X
***Medical Care/ Clinical***				
Medical Health Care Access/ Utilization	X			X
Clinical Measures	X			X
***Accelerometer***				
Use of a wrist-worn accelerometer for one week	X	X	X	X

### Social network survey

Participants’ social networks are measured using egocentric social network (or “personal network”) methods [40]. Participants are asked to name 10 adults whom they consider important in their life and have been in contact with in the past three months. They are asked to provide each social contact’s first name and last initial to protect their privacy. After this, participants are then asked to identify the characteristics of each social contact (also referred to as an “alter”) that they named. They first report on their demographics (gender, age); social role (family member, friend, spouse, coworker, etc.); physical proximity (e.g., if they live in the same house, neighborhood, state, etc.); frequency of contact; emotional closeness they feel with each person; and if the person has been diagnosed with cancer. They also report on their perceptions about whether each social contact does regular physical activity, or if they are typically sedentary. Finally, they report on some of the functional aspects of their relationship with each social contact, including whether or not each social contact provides social support, encourages them to do physical activity, is a barrier to doing physical activity, or is a person with whom they co-engage in physical activity. Lastly, social connections among each of the named social contacts are measured (i.e., who knows whom) to obtain data about the participant’s personal social network structure.

### Statistical methods

#### Data cleaning and computing descriptive statistics

For preliminary analyses, we will screen all data for distributional assumptions (e.g., normality, outliers, multicollinearity) and will perform arithmetic transformations to adjust for skewness or kurtosis if necessary. To handle missing data: during data collection, we attempt to avoid missing data and attrition across the 12-month follow-up. Attrition analyses will be performed to determine whether participants and dropouts differ on any study variables (informative censoring) and whether responders differ from non-responders. If we suspect that missingness is non-random, we will examine the robustness against departures from missing at random (MAR) by using random-coefficient pattern-mixture models, which have been proposed for longitudinal data [41]. Mplus permits construction of structural equation modeling (SEM) that handles missing data using full information maximum likelihood (FIML) methods, in which all available data are included under the MAR assumption to provide unbiased parameter estimates with greater statistical power than listwise deletion [42, 43]. These solutions will be compared to models that handle non-random and random missing data to assess these assumptions.

### Social network analysis

Participants’ personal network data will be analyzed using egocentric social network analysis (SNA) methods. SNA packages in R (e.g., SNA, igraph) will be used to produce visualizations of their personal networks, and compute statistics that summarize the characteristics of each participant’s network, at each survey wave. Characteristics of their *network structure* will be computed based on: (i) *density*, which refers to the number of reported social ties among the alters divided by the total number of possible network ties; (ii) *transitivity*, which reflects the clustering of ties in a social network, and thus the extent that ‘a friend of a friend is a friend’; and (iii) *constraint*, which reflects how connected their alters are to one another and if there are “structural holes” in their networks where groups of alters are not connected, which has been related to the strength of network norms and the diversity of resources to which one has access. Their *network composition* will be measured by calculating the proportion of alters with certain demographic characteristics (e.g., the % of family, same-age peers, presence of a spouse/partner), and the proportion of alters who are physically active and sedentary. Characteristics of their *network functions* will focus on social connection and social support, computed as the proportion of alters that provide specific functions: e.g., the proportion that are close, have frequent contact, provide different measured domains of social support (e.g., generalized social support, support for physical activity), or that are barriers to physical activity. We will conduct exploratory descriptive analyses to understand the types of network members that provide social connection and support; as well as exploratory cluster analyses of respondent network characteristics to identify network structure, composition, and function measures that tend to cluster together (e.g., closeness and support provision). These statistics will be computed for each assessment wave (as well as change statistics across periods, change in the percent of alters that are close, or that provide support), and included in the longitudinal analyses.

### Longitudinal modeling and hypothesis testing

The hypotheses will be tested using multilevel modeling (MLM) within SEM approaches, which can account for the interdependence of repeated observations within participants across the 4-timepoint assessment waves. Covariates will include sex, ethnicity, SES, stage at diagnosis, treatment intensity, age at diagnosis, and current age.

### Trajectories of social health in AYAs

To characterize social health changes over time, we will first test latent growth curve models (LGCM) to identify a general trajectory of social health variables during the course of therapy. A series of latent growth mixture models (LGMM) will be subsequently performed to identify *qualitatively* distinct patterns of social health changes over time.

### Longitudinal associations between social health and activity behaviors in AYAs

To test the association between social health and activity behaviors, which are potentially bidirectional, a two-process parallel latent growth curve model will be fitted, which simultaneously includes growth factors (i.e., initial level and slope) for social health variables and activity behaviors. The parallel process model will be constructed by including: (1) directional paths from baseline social health level to activity behaviors slope as well as baseline activity behaviors level to social health slope; and (2) non-directional correlation paths between social health and activity behaviors (baseline levels, slopes). Furthermore, to test prospective associations of these parallel processes with quality of life outcomes in survivorship, the direct and mediational paths linking growth curves of social health and activity behaviors to quality of life in the SEM will be estimated. If significant indirect paths are detected (e.g., social health → activity behaviors → quality of life), indirect effects will be calculated via Monte Carlo integration methods [44], and the effect sizes will be calculated from the proportion of indirect effect to the total main effect using a protocol of regression-based model [45].

### Effects of sociodemographic and clinical characteristics on the relationship between social health, activity behaviors, and quality of life

To assess the possible moderation effects of demographic and clinical characteristics, multi-group analyses will be conducted by the categories of study moderators (e.g., gender, race/ethnicity, SES, health status). The strengths of group-specific pathways to link social health and activity behaviors to quality of life will be compared using the chi-square (χ2) difference test of model fit. The log-likelihood values with (versus without) the equality constraints on the group-specific pathways will be used to determine if the strength of associations estimated in the models significantly differ by the groups of each moderator (e.g., gender: female vs. male) [46].

### Sample size considerations (power analysis)

Statistical power was tested using procedures of determination of sample size for SEM [47]. Focusing on our hypothesized models, statistical power and minimum sample size estimated by a global fit model index (root mean square error of approximation [RMSEA]) were computed using the R code generated by Preacher and Coffman (2006; http://quantpsy.org/) [48, 49]. Power analysis estimated that the sample of 200 participants would suffice to detect a good to close fit model (RMSEA = .01-.08; α = .05; degrees of freedom [df] = 15–30) with a desired power range from 73% to 80% for detecting moderate to medium path coefficient effect sizes (Cohen’s d = 0.3–0.6) in each of the hypothesized models.

### Study progress

Recruitment for this study commenced February 28, 2022. To date, the study has accrued N = 133 participants who have completed the initial assessment. Of those, N = 62 have completed all four assessment periods. Attrition has been overall low, with 24 patients withdrawing (N = 13), lost to follow-up (N = 9), or deceased before study completion (N = 2). Recruitment will continue into early 2025.

## Discussion

The social health of AYAs has not been comprehensively or prospectively explored in relation to activity behaviors and quality of life in prior research [50]. Therefore, this study will expand our understanding of the mechanisms linking social health to key patient-centered and disease-related health behaviors and outcomes and help identify patients at risk of social impairment. The study methodology will enable a more precise understanding of when, how, and to which patients intervention should occur to prevent declines in social health and its sequelae on activity levels and subsequent quality of life.

One of the most important aspects of this research is the simultaneous assessment of dynamic changes in both individual social experiences and social network characteristics using validated empirical measures. Prior work in this area has focused on more limited measures of social constructs and has often relied on qualitative methods [10]. While such research yields important insights for hypothesis generation, it may fail to discern the distinct social phenomena important to wellbeing outcomes for AYAs. There is, in addition, a pronounced gap in research on how social networks change over time in this population, and how such changes may contribute to changes in social connectivity, social support, and ultimately in activity levels.

Findings from this study have the potential to generate novel and meaningful insights to aid social diagnosing and prescribing and broader social network interventions for this patient population. This can involve tools to identify patients with poor social connections and social health, and strategies to link socially disconnected patients to social and community-based activities that have emerged in primary care and geriatric settings in the United Kingdom and United States with preliminary, yet promising, evidence for effectiveness [51, 52]. Insights from this study could be used to explicitly intervene on AYAs social health to boost their physical activity and wellbeing using age-tailored strategies within the oncology context.

We acknowledge several limitations and strengths of this study. As a single medical system study, our population may be not broadly representative of AYA cancer patients. However, a strength of the study is that USC’s hospital system comprises a comprehensive cancer center, private hospital, and large public safety net hospital which yields diversity in care setting as well in patient sociodemographics. We are also aware of issues regarding potential response bias as patients who are more socially integrated may be more motivated to participate in the study. To address this issue, we work closely with clinicians at USC hospitals to help us reach, build trust with, and encourage the participation of patients who may be less socially integrated and thus less likely to participate. We also work closely with patient advocates who have helped guide the design of appropriate, thoughtful approaches and materials to aid in the recruitment of AYA patients, including those who might otherwise decline to participate. All of these steps help to reduce bias and increase inclusion of diverse patient perspectives.

In summary, this study will address important gaps in our understanding of the impact of a cancer diagnosis on social and physical health outcomes for AYAs. This population is vulnerable to social impairments in addition to persistent treatment-related late effects, health problems due to comorbidities, and poor quality of life. Physical activity, which may alleviate some of this burden of disease, is therefore a priority research area for young survivors who have many life-years ahead of them. Understanding the mechanistic processes by which social health influences activity behaviors and quality of life will lead to the development of more comprehensive and effective interventions to mitigate these challenges.
